# Editorial: Body Representations, Peripersonal Space, and the Self: Humans, Animals, Robots

**DOI:** 10.3389/fnbot.2020.00035

**Published:** 2020-06-16

**Authors:** Matej Hoffmann, Pablo Lanillos, Lorenzo Jamone, Alex Pitti, Eszter Somogyi

**Affiliations:** ^1^Department of Cybernetics, Faculty of Electrical Engineering, Czech Technical University, Prague, Czechia; ^2^Donders Institute for Brain, Cognition and Behaviour, Radboud University, Nijmegen, Netherlands; ^3^ARQ (Advanced Robotics at Queen Mary), School of Electronic Engineering and Computer Science, Queen Mary University of London, London, United Kingdom; ^4^Laboratoire ETIS, CY Cergy Paris University, ENSEA, CNRS, UMR8051, Cergy-Pontoise, France; ^5^Department of Psychology, Centre for Situated Action & Communication, University of Portsmouth, Portsmouth, United Kingdom

**Keywords:** body representations, peripersonal space, self, neurorobotics, cognitive developmental robotics, body schema, body image, development of body representations

The presence of various “body maps” in the brain has fascinated scientists and the general public alike, spurred by the account of Head and Holmes (1911) and the discovery of the somatotopic representations (the “homunculi”) in the primary motor and somatosensory cortices of primates (Leyton and Sherrington, 1917; Penfield and Boldrey, 1937). Neurological conditions and accounts of a whole range of illusions regarding own body perception (e.g., rubber hand illusion, out-of-body experience, apparition) generated both seminal research articles (e.g., Botvinick and Cohen, 1998; Lenggenhager et al., 2007) and public interest. The attention devoted to the representations of the body in the brain has also led to numerous attempts at describing or defining them and proposals of a variety of concepts, such as superficial and postural schema (Head and Holmes, 1911), body schema, body image (Paillard, 1999), corporeal schema, etc. One characteristic common to all these representations is their multimodal nature: they dynamically integrate information from different sensory modalities (visual, tactile, proprioceptive, vestibular, auditory), not excluding motor information (Azañón et al., 2016). However, the concepts of body schema, body image, and many others are umbrella notions for a range of observed phenomena rather than a result of identification of specific mechanisms. The field is thus in a somewhat “chaotic state of affairs” (Berlucchi and Aglioti, 2009), with limited convergence to a common view (Graziano and Botvinick, 2002; Holmes and Spence, 2004). Next to “body space,” the space immediately surrounding the body is called peripersonal space. There are two notions associated with this term: (i) a safety margin around the body, and (ii) space within our reach. They may be supported by distinct neuronal substrates—see Cléry et al. (2015) for a survey. Furthermore, it is not clear to what extent the representation of the “body space” and the space around it are overlapping. They may be “two labels for the same concept” (Cardinali et al., 2009) or rely on a unified representation (Canzoneri et al., 2013). Alternatively, others amass evidence suggestive of their dissociation (Bassolino et al., 2015).

This state of affairs calls for collective action of the interdisciplinary research community and this Research Topic with articles from Frontiers in Psychology—Cognition, Frontiers in Neurorobotics, and Frontiers in Computational Neuroscience is an example of such efforts. Infant development constitutes a key viewpoint from which to study body representations. In our collection, this theme is introduced by Philippe Rochat in his review (Rochat) on self-unity constituting the basis of learning and development. Two original research articles target the somatosensory-motor aspects of early infant development: DiMercurio et al. contribute an observation study of spontaneous touches in the first 2 months of life; Chinn et al. study reaching movements to tactile targets. Tamé et al. also focus on somatoperception—this time in adults. The contributions of Banakou et al., Scarpina et al., Nuara et al., and Arnold et al. deal with plasticity and effects of disorders on body representations. Body representations do not develop in isolation but in a social context—these aspects are studied by Drew et al. in infants, in adults but in a developmental context in Oldroyd et al., Keromnes et al., and in adults, involving a robot to study the effects of anthropomorphism in Heijnen et al.. Peripersonal space as the frontier of self and modulations thereof are reviewed by Cléry and Ben Hamed. Dürr and Schilling study peripersonal space in stick insects.

The remaining contributions employ robots. The motivation is two-fold: First, following the synthetic methodology (“understanding by building”) (Pfeifer and Bongard, 2007; Hoffmann and Pfeifer, 2018), robots can be deployed as embodied computational models of body representations and their development and clear up the notoriously muddy waters of the concepts invented to describe body and self representations. This is the general approach of cognitive developmental robotics and neurorobotics (e.g., Asada et al., 2009) and can be applied to body models specifically (Hoffmann et al., 2010; Schillaci et al., 2016; Lanillos et al., 2017 for surveys). Hafner et al. contribute a conceptual review on the prerequisites for an artificial self. Pugach et al. and Juett and Kuipers present robotic models of peripersonal space representations. Second, the way humans represent their bodies and the space around them provide a proxy for what they expect from a robot collaborator. Hence, a good understanding of these phenomena is the basis for safe and natural human-robot interaction, as studied by Schürmann et al. and also Heijnen et al..

## 1. Infant Development

From the first day of life, newborns manifest awareness of their own body as an invariant and organized spatial structure, coupled with an experiential awareness of the self. Reviewing infancy research of the past few decades, Rochat argues that learning and development rest on this primordial and necessary sense of self-unity. In fact, self-unity, as Rochat proposes, could represent important grounding information for artificial learning systems, allowing them to learn rapidly like human children do.

How does an early sense of the body and self manifest itself in infancy? Two research papers in this section studied the question by examining how infants spontaneously touch their own body and how they reach to tactile targets on the skin. In the first paper, DiMercurio et al. show that infants are active explorers of their own body from the first days of life. In a series of observation sessions, few-week-old infants engaged in a high rate of self-touch, contacting about twenty different areas with each hand, frequently moving from one area to the other. The authors propose that early self-generated and deeply embodied sensorimotor experiences form the critical foundation from which future goal-directed behaviors may develop. Chinn et al. investigated the developmental progression of reaching and grasping strategies to vibrotactile targets attached to various parts of the face. In their longitudinal study, they found that infants are more likely to reach to the target with the hand rather than using other effectors or strategies; they also refine their hand postures with age, using the palmar surface or fingers of the hand rather than the dorsum, and grasping the targets more as they become older.

Young infants not only experience their own bodies but also observe other people's bodies and recognize similarities and differences between them. Such interpersonal aspects of body representations may serve to undergird early social learning. In their EEG study, Drew et al. show that the infant brain registers correspondences between infants' own bodies and the bodies of others. Thus, responses to tactile stimulation to the hand or the foot were modulated by simultaneous vision of the corresponding or non-corresponding effector of another person being touched.

## 2. Adult Body Representations, Plasticity, and Effect of Neurological and Movement Disorders

In their review article, Tamé et al. introduce a new model to describe how tactile processing contributes to a coherent perception of the body as an integrated whole. In a previous model, it was proposed that three types of body representations—the superficial schema, the postural schema, and a model of body size and shape—are required to localize touch in space (Longo et al., 2010). Reviewing evidence, they currently extend this model with two novel dimensions of tactile processing, namely the integration of touch across the two sides of the body and the use of stored proprioceptive information about the location of touch in space (postural priors).

Three clinical research papers examined how the plasticity of the body schema is altered in various neurological conditions. Introducing novel clinical research paradigms based on tool embodiment, graphesthesia tasks, or self-portraits, these articles contribute with valuable results to the relatively scarce existing literature concerning the body schema in patients with Parkinson's disease, somatosensory loss, or cerebral palsy. Scarpina et al. explored how the body schema accommodates significant objects or tools in patients with Parkinson's disease, where motor and sensory bodily functions are primarily affected. Following tool-use training, these patients did not show changes in movement parameters that are associated with effective tool embodiment in healthy individuals. The authors propose that altered plasticity of the body schema is one of the key sensorimotor symptoms in Parkinson's disease. Somatosensory information has a crucial role in self-orientation, as shown in the study by Arnold et al., who examined the effect of somatosensory loss in deafferented patients on the adoption of self-centered vs. decentered perspectives. They compared the responses of two deafferented patients with those of age-matched controls in a graphesthesia task, which consisted of identifying ambiguous tactile letters (such as d and b) drawn on various surfaces of the head. Deafferented patients relied on individual cognitive strategies and responded with greater variability across head and trunk orientation conditions. On the other hand, the control group, consistent with earlier studies, reliably adopted self-centered perspectives for tactile letters drawn on the forehead or on side surfaces of the head which were aligned with the front surface of the trunk. How do representations of self and body develop in children with a neurological condition, such as unilateral cerebral palsy? Using self- and peer portraits, Nuara et al. report evidence that body self-representation—more specifically the children's own experience with their body's functioning—is reflected in their drawings.

Finally, Banakou et al. explored whether our cognitive performance, attitudes, and perhaps behaviors also change when we switch bodies in virtual reality settings. The plasticity of our body schema allows us to easily perceive a virtual life-sized body as our own even when the virtual body is strikingly different from our own. They report exciting results showing that virtual embodiment—adopting the body of Albert Einstein in this case—can cause changes in cognitive processing and also a reduction in age-based discrimination of young adults toward the elderly.

## 3. Social Aspects of Body Representations

Alongside early sensorimotor experiences, early social experiences also have a substantial impact on the areas of the brain responsible for representation of the body. Oldroyd et al. explored how attachment between child and caregiver might be linked to interoception—an individual's ability to detect and track internal bodily cues. They found that an avoidant attachment style was associated with lower interoceptive functioning, whereas an anxious attachment style was associated with heightened interoception. Furthermore, reported parenting was associated with youths' awareness of their physiological and emotional responding.

Keromnes et al. present a historical review of the concept of self-consciousness and provide an overview of the role of body perception in the construction of a sense of self as well as the differentiation of self and other. They demonstrated that a multidisciplinary approach is mandatory to address such a complex concept. The paper highlights the importance of self-image recognition in the mirror to assess self-consciousness but also the role of the other in self-image recognition. Self-image development might be a good indicator of the evolution of self-consciousness.

Heijnen et al. analyzed the impact of movement synchronization on the level of anthropomorphization of a robot. Two competing hypotheses were behind the study: (1) feature overlap, i.e., self-other overlap, will activate features related to humans; (2) autonomy, where unpredictability (unsynchronized condition) will increase anthropomorphization. Results did not show any significant influence of the synchronization manipulation regarding the attributed anthropomorphization of the robot.

## 4. Peripersonal Space Representations and Robotic Models Thereof

Two articles from the collection deal with the space around the body. Cléry and Ben Hamed in their review summarize recent neuroscience research on peripersonal space (PPS) representations, focusing both on PPS models of individual body parts (e.g., hand, face, trunk) and models of their interaction, and suggesting possible avenues for future studies. The paper discusses how visual and tactile events in the PPS are predicted (both temporally and spatially), how the PPS is modulated (for example, by tool use, by other perceptual stimuli, by social factors), what is the relationship between PPS and Interpersonal Space, and how individual personality traits can affect the PPS. Ultimately, the links between PPS and bodily self-consciousness are discussed. Dürr and Schilling propose a formalization of PPS in insects, and in particular offer a description of how the PPS of a stick insect (*Carausius morosus*) would look like. Whole-body motion capture data of unrestrained walking, climbing and searching behaviors is used to delineate “action volumes” and “contact volumes” for both antennae and all six legs of the insect; the intersection of these volumes is equivalent to a representation of coinciding somatosensory and motor activity, and can therefore be representative of the PPS. Then, overlapping regions of the action spaces of each pair of limbs are deducted and referred to as affordance space, which defines regions of the space in which the motion of one limb influences the possible motion of another limb. Finally, an artificial neural network model is proposed to model the motion interaction between pair of limbs, based on the aforementioned affordance space.

Two articles employ robotic models to study PPS-related phenomena. Pugach et al. propose a neural model based on Gain-Field neurons for integrating tactile events with arm postures and visual locations for constructing hand- and target-centered receptive fields in the visual space. In robotic experiments using an artificial skin, they show how their neural architecture reproduces the behaviors of parietal neurons for: (1) dynamically encoding the body schema of a robotic arm without any visual tags on it, and (2) estimating the relative orientation and distance of targets to it. By doing so, they demonstrate how tactile information facilitates the integration of visual and proprioceptive signals in order to construct the body space. Juett and Kuipers present a computational model that enables a robot to automatically build a representation of its peripersonal space (PPS) by sensorimotor exploration. Following a developmental approach based on intrinsic motivation, the robots first performs motor babbling and begins to discover patterns of regularities and unusual events in the sensorimotor (visuomotor) space; gradually, this leads to the emergence of goal-directed reaching and grasping abilities. Preliminary results obtained with a Baxter bimanual robot support the validity of this approach, and its applicability to real-world situations.

## 5. Human-Like Body Models in Robots

Finally, two articles from researchers in robotics provide bridges between the body representations in biology and machine body models. Hafner et al. discuss the minimal requirements for a robot to develop an artificial sense of self. For a *minimal self* , they focus on sense of body ownership and agency and analyze how an artificial agent could develop these capacities and how that could be measured. Self-exploration behaviors, artificial curiosity, sensorimotor simulations, and predictive processes are discussed in this context. Schürmann et al. in their perspective article take a more pragmatic, application-oriented approach and discuss how taking inspiration in biological body representations can be exploited in assistive devices and humanoid robots.

## Author Contributions

All authors listed have made a substantial, direct and intellectual contribution to the work, and approved it for publication.

## Conflict of Interest

The authors declare that the research was conducted in the absence of any commercial or financial relationships that could be construed as a potential conflict of interest.
